# DNA-Dependent Protein Kinase As Molecular Target for Radiosensitization of Neuroblastoma Cells

**DOI:** 10.1371/journal.pone.0145744

**Published:** 2015-12-30

**Authors:** M. Emmy M. Dolman, Ida van der Ploeg, Jan Koster, Laurel Tabe Bate-Eya, Rogier Versteeg, Huib N. Caron, Jan J. Molenaar

**Affiliations:** 1 Department of Oncogenomics, Academic Medical Center, University of Amsterdam, Amsterdam, the Netherlands; 2 Department of Pediatric Oncology, Emma Kinderziekenhuis, Academic Medical Center, University of Amsterdam, Amsterdam, the Netherlands; St. Georges University of London, UNITED KINGDOM

## Abstract

Tumor cells might resist therapy with ionizing radiation (IR) by non-homologous end-joining (NHEJ) of IR-induced double-strand breaks. One of the key players in NHEJ is DNA-dependent protein kinase (DNA-PK). The catalytic subunit of DNA-PK, i.e. DNA-PKcs, can be inhibited with the small-molecule inhibitor NU7026. In the current study, the *in vitro* potential of NU7026 to radiosensitize neuroblastoma cells was investigated. DNA-PKcs is encoded by the *PRKDC (protein kinase*, *DNA-activated*, *catalytic polypeptide)* gene. We showed that *PRKDC* levels were enhanced in neuroblastoma patients and correlated with a more advanced tumor stage and poor prognosis, making DNA-PKcs an interesting target for radiosensitization of neuroblastoma tumors. Optimal dose finding for combination treatment with NU7026 and IR was performed using NGP cells. One hour pre-treatment with 10 μM NU7026 synergistically sensitized NGP cells to 0.63 Gy IR. Radiosensitizing effects of NU7026 increased in time, with maximum effects observed from 96 h after IR-exposure on. Combined treatment of NGP cells with 10 μM NU7026 and 0.63 Gy IR resulted in apoptosis, while no apoptotic response was observed for either of the therapies alone. Inhibition of IR-induced DNA-PK activation by NU7026 confirmed the capability of NGP cells to, at least partially, resist IR by NHEJ. NU7026 also synergistically radiosensitized other neuroblastoma cell lines, while no synergistic effect was observed for low DNA-PKcs-expressing non-cancerous fibroblasts. Results obtained for NU7026 were confirmed by *PRKDC* knockdown in NGP cells. Taken together, the current study shows that DNA-PKcs is a promising target for neuroblastoma radiosensitization.

## Introduction

The DNA damage response plays a dual role in cancer since it prevents genomic instabilities that can cause cancer, while on the other hand it might protect tumors from therapy-induced DNA damage [1–3]. Under normal circumstances, cells have a variety of repair pathways for the repair of DNA single- and double-strand breaks (SSBs and DSBs) to maintain genomic stability [4]. DNA DSBs are in general very destructive and are primarily restored by non-homologous end-joining (NHEJ) or homologous recombination (HR). The choice between NHEJ and HR depends on the nature of the DNA damage and the cell cycle stage of the cells [5, 6]. NHEJ is the major DSB repair pathway and is active in all phases of the cell cycle, while HR is only active in the S/G2 phase of the cell cycle. Broken DNA ends are directly ligated in NHEJ, without the presence of a homologous sequence [6–8]. DNA-dependent protein kinase (DNA-PK), consisting of the DNA end-binding heterodimer Ku70/80 and the catalytic subunit DNA-PKcs, plays a key role in NHEJ. It recognizes DSBs, facilitates DNA ligation and recruits and activates proteins that are responsible for the processing and final ligation of the broken DNA ends [9–12].

Many therapeutic strategies applied in cancer treatment, including ionizing radiotherapy, aim to kill cancer cells by inducing DNA damage [12]. Restoration of damaged DNA by the DNA damage response then might result in decreased compound efficacy or resistance [13, 14]. Resistance of cancer cells to radiotherapy has been observed for different types of cancer, including neuroblastoma [15–17]. High-risk neuroblastoma patients are often treated with external beam radiotherapy for the primary tumor and in some protocols with^131^I-MIBG (metaiodobenzylguanidine) prior to chemotherapy. A promising strategy to radiosensitize neuroblastoma cancer cells and overcome therapy resistance is to combine radiotherapy with a DNA repair inhibitor that selectively interferes in the repair cascade [12, 13, 18, 19]. This strategy might even be more efficacious when the activity of the targeted DNA repair pathway is elevated in cancer and cancer cells are depending on the higher levels of these proteins. Ionizing radiation (IR) destroys tumor cells by inducing lethal DSBs, among other DNA damaging effects [12, 18]. Since these IR-induced DSBs can be repaired by DNA-PK-mediated NHEJ [20], co-administration of a DNA-PK inhibitor might be advantageous.

Several small-molecule inhibitors of DNA-PKcs are developed, which have successfully been evaluated *in vitro* for their potency to sensitize cells to chemo- and radiotherapy [21–28]. Among these inhibitors is the selective ATP-competitive DNA-PKcs inhibitor NU7026, which has been shown to radiosensitize ovarian, pancreatic and gastric tumor cells *in vitro* [29–31].

The current paper shows that DNA-PKcs is a promising target for neuroblastoma radiosensitization, as low radiation doses might be used in combination with NU7026 and this combination does not affect low DNA-PKcs expressing cells.

## Materials and Methods

### Chemicals

NU7026 and gemcitabine were purchased from TOCRIS Bioscience (Bristol, UK) and Sigma Aldrich (Zwijndrecht, the Netherlands), respectively. Stock solutions of 10 mM were prepared in DMSO (NU7026) and demineralised water (gemcitabine) and aliquots were stored at -20°C till further use.

### Affymetrix microarray analysis

Affymetrix microarray analyses were performed to determine *PRKDC* mRNA expression levels in neuroblastoma cell lines and a neuroblastic tumor panel consisting of 88 neuroblastoma samples derived from primary tumors of untreated patients [32]. Patient material was obtained during surgery and immediately frozen in liquid nitrogen. Total RNA isolation, Affymetrix mRNA profiling and data analysis were performed as described previously [33]. The Affymetrix microarray profiling results for the cohort of 88 neuroblastoma tumors have been deposited at the Gene Expression Omnibus under accession GSE16476. As a second neuroblastic tumor panel, the public available dataset of Delattre was used [34]. Affymetrix expression data from normal tissues in adults were derived from the Expression Project for Oncology database from the International Genomics Consortium.

### Cell culture

Human fibroblast lines F1366, F1641, F0577 and F2112 were isolated from patient materials under informed consent at the department of oncogenomics from the university of Amsterdam (the Netherlands), as previously described [35, 36]. Human neuroblastoma cell lines (NGP, SHSY5Y, SHEP2, SJNB12, LAN5 and SKNBE(2)) [37] and fibroblast lines were grown in Dulbecco’s modified Eagle’s medium (DMEM) containing 4.5 g/L D-glucose, glutamate and supplemented with 10% (v/v) foetal calf serum, 2 mM L-glutamine, 10 U/mL penicillin, 10 μg/mL streptomycin and MEM non-essential amino acids (1x). Cells were maintained at 37°C under 5% CO_2_ in humidified air. Penicillin and streptomycin were obtained from Sigma Aldrich, all other cell culture related materials were obtained from PAA Laboratories GmbH (Pasching, Austria).

### Lentiviral short hairpin RNA production and transduction optimization

Lentiviral particles were produced in HEK293T cells by transduction of lentiviral vector containing the short hairpin RNA (shRNA) with lentiviral packaging plasmids pMD2G, pRRE, and pRSV/REV using FuGene HD. Supernatant of the HEK293T cells was harvested at 48 and 72 h after transduction, and purified by filtration and ultracentrifugation. Cells were seeded at 10–20% confluence. After 24 h cells were transduced with lentiviral *PRKDC* shRNA (Sigma, TRCN0000197152) in various volumes (5, 10, 20, 40 and 80 μL). SHC002 shRNA (non-targeting shRNA: AACAAGATGAAGAGCACCAA) was used as a negative control. Twenty-four hours after transduction, medium was refreshed and puromycin (1 μg/mL) was added to determine the efficacy of transduction. Protein was harvested 48 h after transduction and used for Western Blot analysis of DNA-PKcs to determine the volume required for the optimal transduction efficiency and knockdown.

### *PRKDC* knockdown

NGP cells were seeded in 6-cm culture dishes at various seeding densities and transduced after 24 h with *PRKDC* shRNA or a non-targeting shRNA control (SHC002). After another 24 h, medium was refreshed by medium supplemented with puromycin. Knockdown of *PRKDC* mRNA and DNA-PKcs protein levels was confirmed by Q-PCR (144 h after transduction) and Western Blot analysis (168 h after transduction), respectively. For target validation studies, cells were exposed to 0.63 Gy IR at 48 h after transduction. The occurrence of apoptosis was studied at indicated time points after IR-exposure by Western Blot and FACS analysis.

### Q-PCR

Total RNA of NGP cells transduced with *PRKDC* shRNA or SHC002 was extracted using Trizol reagent (Invitrogen, Breda, the Netherlands) according to the manufacturers protocol. RNA concentration was determined using the NanoDrop ND-1000. cDNA was prepared using 1 μg of total RNA, 125 pmol oligo(dT)12 primer, 0.36 mM deoxyribonucleotide triphosphates, 1.4 mM MgCl_2_, 10x Reverse Transcriptase buffer and 200 U/μL superscript III in a total volume of 35 μL (Invitrogen). One microliter cDNA was used for Q-PCR. A fluorescence based kinetic real-time PCR was performed using the real-time iCycler PCR platform in combination with the intercalating fluorescent dye IQ SYBR green supermix (Bio-Rad, Veenendaal, the Netherlands). The IQ SYBR green I supermix was used according to the manufacturers instructions. Following primers were used: β-actin forward: CCCAGCACAATGAAGATCAA reverse: ACATCTGCTGGAAGGTGGAC, *PRKDC* forward: TGCAGCTGATTCACTGGTTC reverse: TCGAATACACCGACCACAAA.

### Irradiation

To induce DSBs, an external Caesium 137 (^137^Cs) gamma irradiator was used. Cells were exposed to various amounts of ionizing radiation (IR) as described.

### Optimal dose finding for combination treatment

Evaluation of the most optimal drug-IR combinations has been performed in NGP cells because of their high *PRKDC* mRNA expression. NGP cells were seeded at a 20% confluence in 96-well plates and cultured overnight. For the combination of NU7026 and IR, cells were pre-incubated with NU7026 in a dose range of 0–50 μM for 1 h under normal culture conditions and subsequently exposed to IR in a dose range of 0–6.25 Gy. NU7026 was left in the culture medium and cell viability was established at 48, 72 and 96 h after IR-exposure. For the combination of gemcitabine and IR, NGP cells were pre-incubated with gemcitabine in a dose range of 0–10 μM for 3 or 24 h and subsequently exposed to 0.63 Gy IR. Cell viability was established at 96 h after IR-exposure. Most optimal drug-IR combinations were used for further analyses.

### MTT cell proliferation assay

Cell viability was established at indicated time points using the 3-(4,5-dimethylthiazol-2-yl)-2,5-diphenyltetrazolium bromide (MTT) colorimetric assay [38]. Statistical analysis was performed using one-way analysis of variance (ANOVA), with p<0.05 as the minimum level of significance.

### Soft agar colony formation assay

Anchorage-independent soft agar colony formation assays on NGP cells were performed in 24-well plates. First, base layers of 0.5% (w/v) agar in culture medium were prepared (250 μL/well). After solidification, single cell suspensions in 0.4% agar in culture medium (20000 cells/mL) were prepared and cells were added on top of the base layers (5000 cells in 250 μL/well). After overnight incubation at normal culture conditions, cells were 1 h pre-incubated with 0, 2, 10 or 20 μM NU7026 by adding 500 μL 0.4% agar in culture medium supplemented with DMSO or NU7026. Next, cells were exposed to 0 or 0.63 Gy IR. The following 3 weeks, 500 μL/well fresh 0.4% agar in culture medium supplemented with DMSO or NU7026 was added to the cells once a week. Colonies were visualized by 4 h incubation with 250 μL 1 mg/mL MTT in PBS.

### FACS analysis

Attached and floating cells were fixated with 100% ethanol at -20°C. Cells were stained with 0.05 mg/mL propidium iodide and 0.05 mg/ml RNAse A in PBS. After 1 h incubation in the dark at room temperature (RT), DNA content of the nuclei was analyzed using a fluorescence-activated cell sorter. A total of 20,000 nuclei were counted per sample. The cell cycle distribution and the apoptotic sub-G1 fraction were determined using the Accuri^TM^ C6 Flow Cytometer with CFlow Plus software (BD Biosciences, Erembodegem, Belgium).

### Western Blot analysis

Cells were lysed at indicated time points using Laemmli buffer (i.e. H_2_O/glycerol/20% sodium dodecyl sulphate (SDS)/1 M Tris-HCl (pH 6.8) 5:2:2:1 (v/v/v/v)) supplemented with sodium fluoride and sodium vanadate (final concentration 0.5 mM). Protein concentrations were determined using the Bio-Rad *DC* Protein Assay. Equal protein amounts were diluted with 5x reducing sampling buffer (i.e. Laemmli buffer/β-mercaptoethanol 3:1 (v/v) with bromophenol blue sodium salt). Diluted samples were boiled (5 min; 95°C) and centrifuged (13000 rpm; 2 min). Proteins were separated by SDS-polyacrylamide gel electrophoresis on 10% (DNA-PKcs and cleaved PARP) or 12% (p-DNA-PKcs and β-actin) Mini-Protean^®^ Tris-glycince extended precast gels (Bio-Rad). Seperated proteins were transferred onto Immobilon^®^-FL polyvinylidene fluoride (PVDF) membranes by overnight wet blotting at 30 V. Transfer buffer consisted of 20% (v/v) methanol, 3.025 g/L Tris and 14.4 g/L glycine in demineralized water.

For β-actin and α-tubulin, membranes were blocked with Odyssey blocking buffer (OBB) (LI-COR, Westburg BV, Leusden, the Netherlands) 1:1 diluted in PBS (1 h; RT). After blocking, membranes were incubated with mouse anti-human β-actin (clone AC-15) monoclonal antibody (1:20000; Abcam) or mouse anti-human α-tubulin (clone DM1A) monoclonal antibody (1:10000) in OBB with 0.1% (v/v) Tween-20 (OBB-T) (overnight; 4°C). Next, membranes were incubated with IRDye 800CW goat anti-mouse secondary antibody (1:5000; Li-COR) in OBB-T (1 h; RT) and visualized on the LI-COR Odyssey.

For cleaved PARP, membranes were blocked with 5% (w/v) nonfat dry milk in Tris-buffered saline (TBS) (1 h; RT) and subsequently incubated with mouse anti-human PARP monoclonal antibody (1:1000; BD Biosciences) in TBS containing 0.1% (v/v) Tween-20 (TBS-T) (overnight; 4°C). After incubation with IRDye 800CW goat anti-mouse secondary antibody (1:5000; Li-COR) in TBS-T (1 h; RT), membranes were visualized on the LI-COR Odyssey.

For DNA-PKcs and p-DNA-PK, membranes were blocked with 5% (w/v) nonfat dry milk in PBS containing 0.1% (v/v) Tween-20 (PBS-T, 1 h; RT) and subsequently incubated with rabbit anti-human DNA-PKcs (clone Y393) monoclonal antibody (1:1000; Abcam, Cambridge, UK) or rabbit anti-human p-DNA-PK (S2056) polyclonal antibody (1:400; Abcam) in 5% (w/v) bovine serum albumin (BSA) in PBS-T (1 h; RT). Membranes were then incubated with Amersham horseradish peroxidase (HRP)-conjugated ECL^TM^ donkey anti-rabbit secondary antibody (1:5000; GE Healthcare, Hoevelaken, the Netherlands) (1 h; RT). Proteins were visualized by a chemiluminescence-based detection reagent (SuperSignal^®^ West Femto; Thermo Fischer Scientific, Etten-Leur, the Netherlands) and band density was determined on the ImageQuant LAS 4000 mini (GE Healthcare, Diegem, Belgium).

## Results

### Enhanced *PRKDC* expression in neuroblastoma patients correlates with poor prognosis

**Fig 1:**
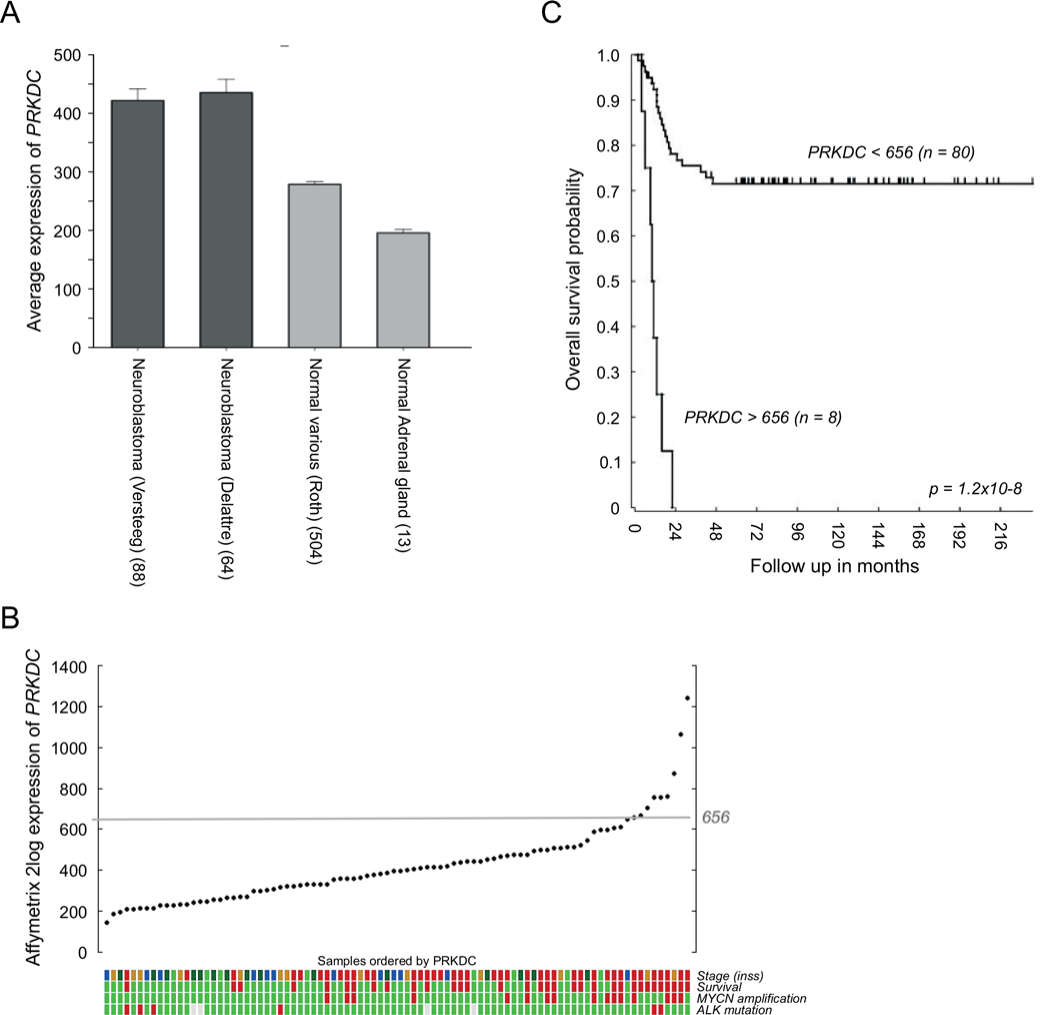
Enhanced *PRKDC* expression in neuroblastoma patients correlates with a poor prognosis. (A) Comparison between the average Affymetrix *PRKDC* mRNA expression levels in two independent neuroblastoma datasets (dark grey) versus expression levels in a compiled library of normal tissues and in nonmalignant adrenal glands (light grey). (B) Affymetrix *PRKDC* mRNA expression in neuroblastoma of 88 individual patients, ordered by the ^2^log fold transformed level of *PRKDC*. Color coded tracks below the YY plot give information about neuroblastoma stage (INSS; green = stage 1, dark green = stage 2, light brown = stage 3, blue = stage 4s and red = stage 4), patient survival (green = yes and red = no) and the presence of *MYCN* amplification and *ALK* mutation (green = yes and red = no). (C) Kaplan-Meier curve of the survival of neuroblastoma patients with an Affymetrix *PRKDC* mRNA expression above 656 (n = 8) versus neuroblastoma patients with an Affymetrix *PRKDC* mRNA expression below 656 (n = 80). The cutoff of 656 was determined using the Kaplan scanner tool in the R2 web application (http://r2.amc.nl). Samples were sorted according to the expression of *PRKDC* and divided into two groups on the basis of a cutoff expression value. All cutoff expression levels and the resulting groups were analyzed for survival, with the provision that each group included at least eight samples. For each cutoff level and grouping, the log-rank significance of projected survival was calculated. The graph depicts the best *p* value corrected for multiple testing (Bonferroni correction).

For clinical purposes it would be desirable that anticancer agents exert their effects specifically on cancer cells, so unwanted side effects in healthy organs will be limited. We therefore evaluated *PRKDC* mRNA expression levels in human neuroblastoma patients and compared these expression levels with expression levels in non-diseased (normal) tissues. As shown in Fig 1A, *PRKDC* mRNA expression levels in two independent neuroblastoma tumor datasets were significantly higher than the expression levels in a compiled library of normal tissues and nonmalignant adrenal glands (the main primary tumor site) [39]. Elevated *PRKDC* mRNA levels in neuroblastoma were associated with poor clinical characteristics (Fig 1B) and a significant reduction in overall survival (Fig 1C).

### NU7026 sensitizes NGP cells to low IR doses

**Fig 2:**
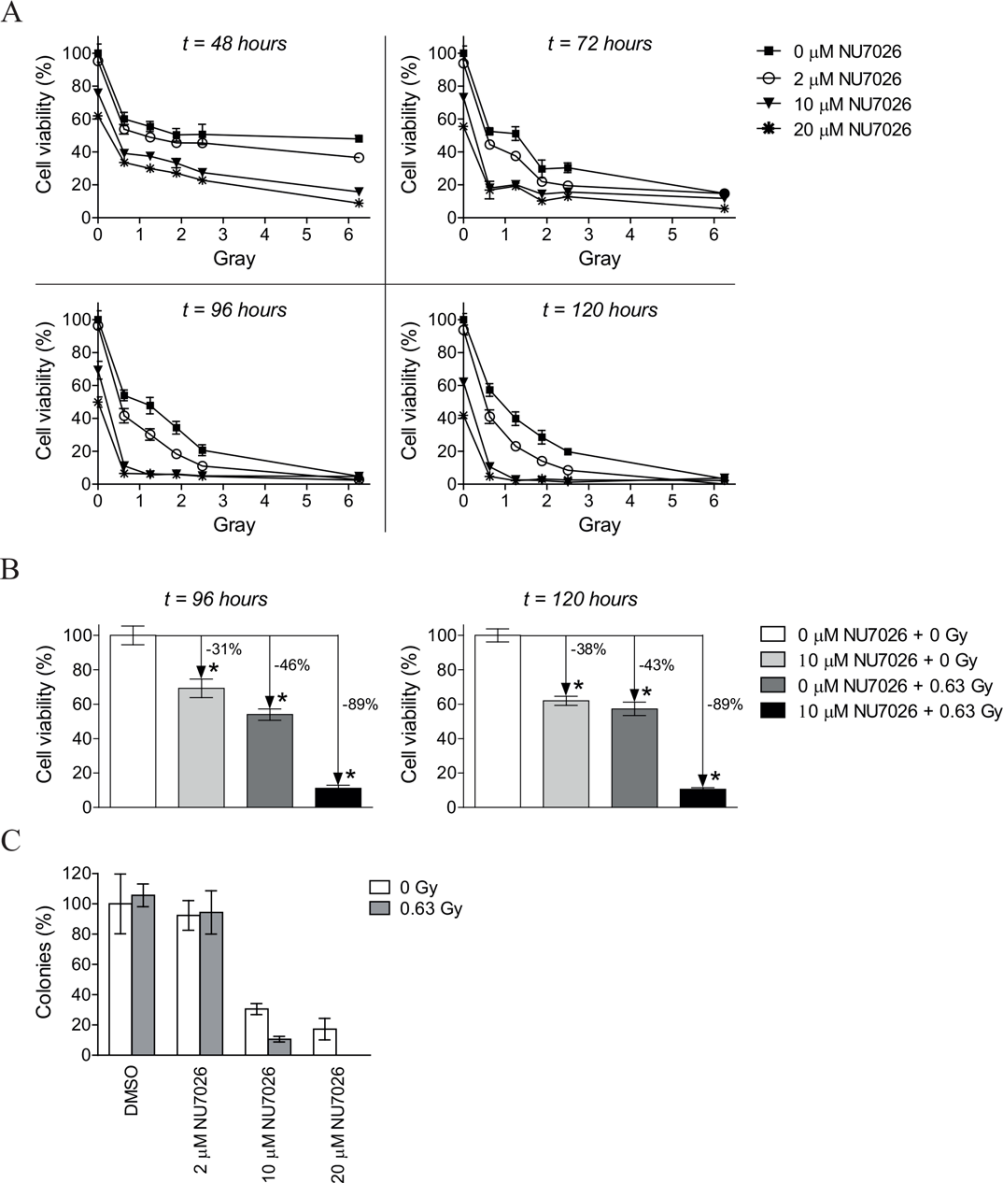
DNA-PKcs inhibitor NU7026 radiosensitizes NGP cells to low IR doses. (A) Cell viability of NGP cells after co-treatment with NU7026 and IR. Cells were treated with 0, 2, 10 or 20 μM NU7026, 1 h prior to exposure to up to 6.25 Gy IR. At 48, 72, 96 and 120 h after IR-exposure, MTT cell proliferation assays were performed to study inhibitory effects on cell viability. Data represent the mean (n = 4) +/- SD. (B) Cell viability of NGP cells after treatment with 10 μM NU7026 and/or 0.63 Gy IR at 96 and 120 h after IR-exposure. Data represent the mean (n = 4) +/- SD. Statistical differences between untreated and treated NGP cells are indicated as * (*p*<0.05). (C) Effects of NU7026 plus IR combination therapy versus monotherapy on the colony forming capacity of NGP cells. Cells in 0.4% agar in culture medium were seeded on top of a hardened 0.5% agar base layer. After overnight incubation at normal culture conditions, cells were 1 h pre-incubated with 0, 2, 10 or 20 μM NU7026 in 0.4% agar in culture medium before exposure to 0 or 0.63 Gy IR. The following 3 weeks, fresh DMSO or NU7026 in 0.4% agar in culture medium was added to the cells once a week. Colonies were subsequently visualized by 4 h incubation with MTT and counted. The number of colonies formed by untreated NGP cells has been set at 100%. Data represent the mean (n = 3) +/- SD.

When combining a radiosensitizer with radiotherapy it would be most favorable when monotherapy with either of the therapeutics alone does not result in major effects, while a maximum anticancer activity is obtained after combination treatment. Optimal doses for combination treatment with NU7026 and IR were studied in the neuroblastoma cell line NGP because of the high *PRKDC* mRNA expression in this cell line, as determined by Affymetrix microarray analysis (S1 Fig). Fig 2 shows the cell viability of NGP cells pre-treated with 0, 2, 10 or 20 μM NU7026 before being exposed to increasing doses of IR up to 6.25 Gy. Effects on cell viability after combination treatment with NU7026 and IR increased in time, with maximum effects observed from 96 h after IR-exposure on (Fig 2A). As anticipated, cytotoxic effects of monotherapy with NU7026 or IR increased with increasing dose (S1 Table).

Combination treatment of NGP cells with 10 μM NU7026 and 0.63 Gy IR resulted in a decrease in cell viability of 89% (at 96 as well as 120 h after IR-exposure), while maximum inhibitory effects achieved with 10 μM NU7026 and 0.63 Gy IR monotherapy were no more than 38 and 46%, respectively (Fig 2B). To quantify the interaction between 10 μM NU7026 and 0.63 Gy IR, combination indices (CIs) were determined according to Chou and Talalay [40]. From these CIs (i.e. 0.25 and 0.26 at 96 and 120 h after IR-exposure, respectively) it could be concluded that synergistic radiosensitization of NGP cells was obtained. CIs for all combinations are presented in S1 Table.

Radiosensitizing effects of NU7026 were confirmed by analysis of the long-term effects on colony formation. In line with the effects on cell viability, NU7026 monotherapy dose-dependently inhibited the colony forming capacity of NGP cells (Fig 2C and S2A Fig). While irradiation with 0.63 Gy alone did not result in any effect, pre-treatment of NGP cells with 10 or 20 μM NU7026 before irradiation resulted in less colonies compared with NU7026 monotherapy. Further combination studies were performed using 10 μM NU7026 and 0.63 Gy IR.

### Combination treatment with NU7026 and IR induces apoptosis of NGP cells

**Fig 3:**
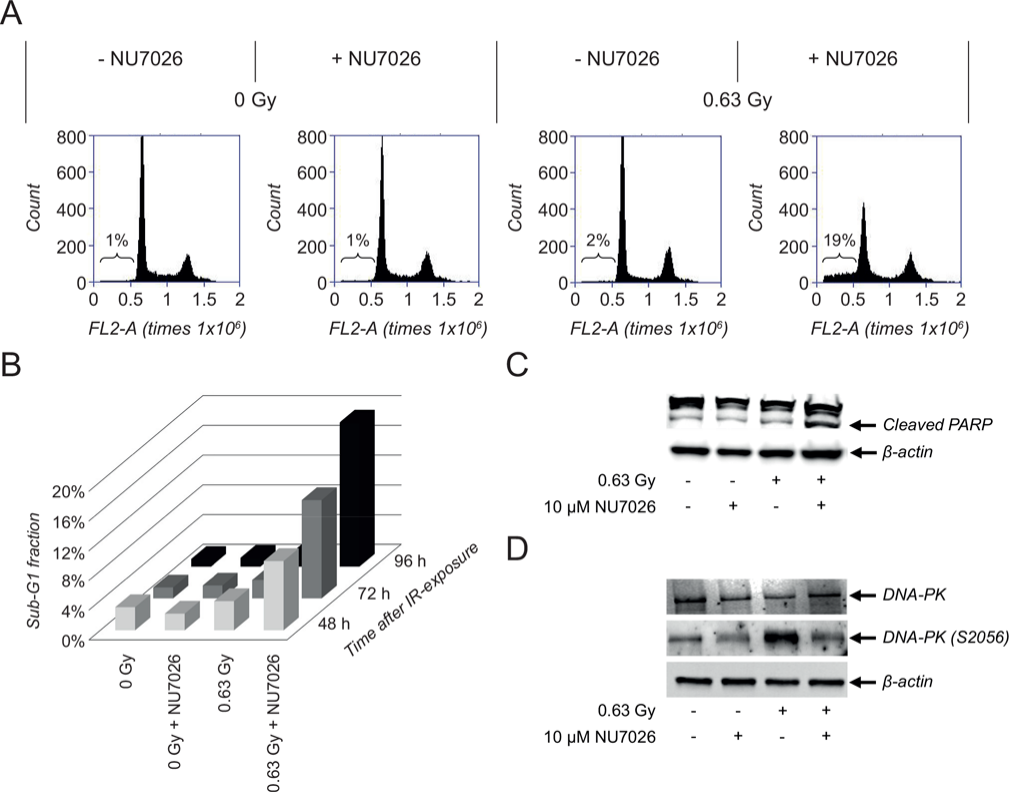
Combination treatment with NU7026 and IR induces apoptosis in NGP cells. (A) Effects of NU7026 plus IR combination therapy versus monotherapy on the cell cycle status of NGP cells. For combination treatment, cells were pre-treated with 10 μM NU7026 for 1 h before exposure to 0.63 Gy IR. Samples were analyzed by FACS analysis at 96 h after treatment. (B) FACS analysis of the effects of combination therapy versus monotherapy on the sub-G1 fraction of cells at 48, 72 and 96 h after treatment. (C) Western Blot detection of total DNA-PKcs and activated DNA-PKcs (via phosphorylation at serine 2056) levels and PARP cleavage after combination therapy versus monotherapy with 10 μM NU7026 and/or 0.63 Gy IR, at 1 h and 96 h after IR-exposure, respectively. β-actin protein levels were used as loading control.

As the cell viability and colony forming responses shown in Fig 2 represent combined effects on cell proliferation and cell death, additional analyses were performed to study specific effects on apoptosis. Combination treatment of NGP cells with 10 μM NU7026 and 0.63 Gy IR resulted in a remarkable increase in sub-G1 fraction (i.e. + 18% versus untreated cells), while hardly any effect was obtained after monotherapy with NU7026 or IR (i.e. + 0% and 1% versus untreated cells, respectively) (Fig 3A and 3B). Most strikingly, the synergistic effect between NU7026 and IR is much more pronounced for the apoptotic response than for the cell viability response shown in Fig 2. Observed effects on sub-G1 fraction increased in time (Fig 3B), confirming the time-dependent effects on cell viability. Apoptosis of co-treated NGP cells was additionally shown by PARP cleavage at 96 h after IR-exposure (Fig 3C).

DNA-PKcs was rapidly activated upon cell exposure to 0.63 Gy IR, as shown by the increase in DNA-PKcs autophosphorylation at serine 2056 observed at 1 h after irradiation. IR-induced DNA-PKcs activation was inhibited by concomitant treatment with 10 μM NU7026, added to the cells at 1 h prior to IR-exposure (Fig 3D). These results confirm that NGP cells can partially evade cytotoxicity of IR by activating NHEJ.

### NU7026 does not radiosensitize low DNA-PKcs-expressing non-cancerous fibroblasts

**Fig 4:**
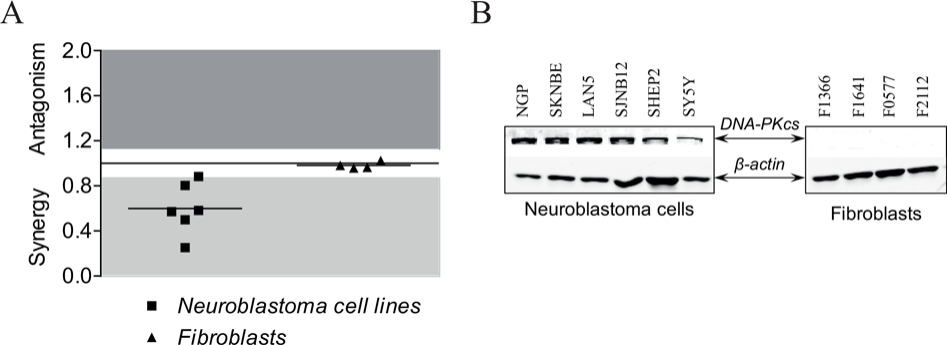
NU7026 does not radiosensitize low DNA-PKcs-expressing non-cancerous cells. (A) Radiosensitizing effect of NU7026 in neuroblastoma cell lines NGP, SHSY5Y, SHEP2, SJNB12, LAN5 and SKNBE(2) compared with radiosensitizing effects in non-cancerous fast-proliferating fibroblast cell lines F1366, F1641, F0577 and F2112. Co-treated cells were exposed to 0.63 Gy IR after 1 h pre-treatment with 10 μM NU7026. At 96 h after IR-exposure, MTT cell proliferation assays were performed to assess cell viability (n = 4 for each cell line). Radiosensitizing effects were estimated by calculating combination indices (CI) according to Chou and Talalay [40], given on the Y-axis. CI > 1.1 is antagonistic (dark grey), 1.1 ≥ CI ≥ 0.9 is additive (white) and CI < 0.9 is synergistic (light grey). Horizontal lines between the symbols represent the mean combined effect obtained for all neuroblastoma- or fibroblast cell lines. (B) Western Blot analysis of DNA-PKcs protein levels in neuroblastoma cell lines NGP, SHSY5Y, SHEP2, SJNB12, LAN5 and SKNBE(2) and fibroblast cell lines F1366, F1641, F0577 and F2112. β-actin protein levels were used as loading control.

Radiosensitizing effects of NU7026 were investigated in five additional neuroblastoma cell lines (i.e. SHSY5Y, SHEP2, SJNB12, LAN5 and SKNBE(2)) with varying *PRKDC* mRNA expression levels (S1 Fig). As shown in Fig 4A, combination treatment with NU7026 and IR synergistically inhibited cell viability in all tested neuroblastoma cell lines, with CIs (calculated at 96 h after IR-exposure) varying from 0.25 to 0.88. Radiosensitizing effects for neuroblastoma cell lines were subsequently compared with the radiosensitizing effects of NU7026 for a panel of non-cancerous fast-proliferating fibroblasts (i.e. F1366, F1641, F0577 and F2112) (Fig 4A). In non-cancerous fibroblasts an additive inhibitory effect on cell viability of NU7026 in combination with IR was observed, with CIs between 0.96 and 1.02. This differential effect might be explained by up-regulated DNA-PKcs protein levels in neuroblastoma cells, as only limited levels of DNA-PKcs could be detected in the fibroblast cell lines (Fig 4B and S2B Fig).

**Fig 5:**
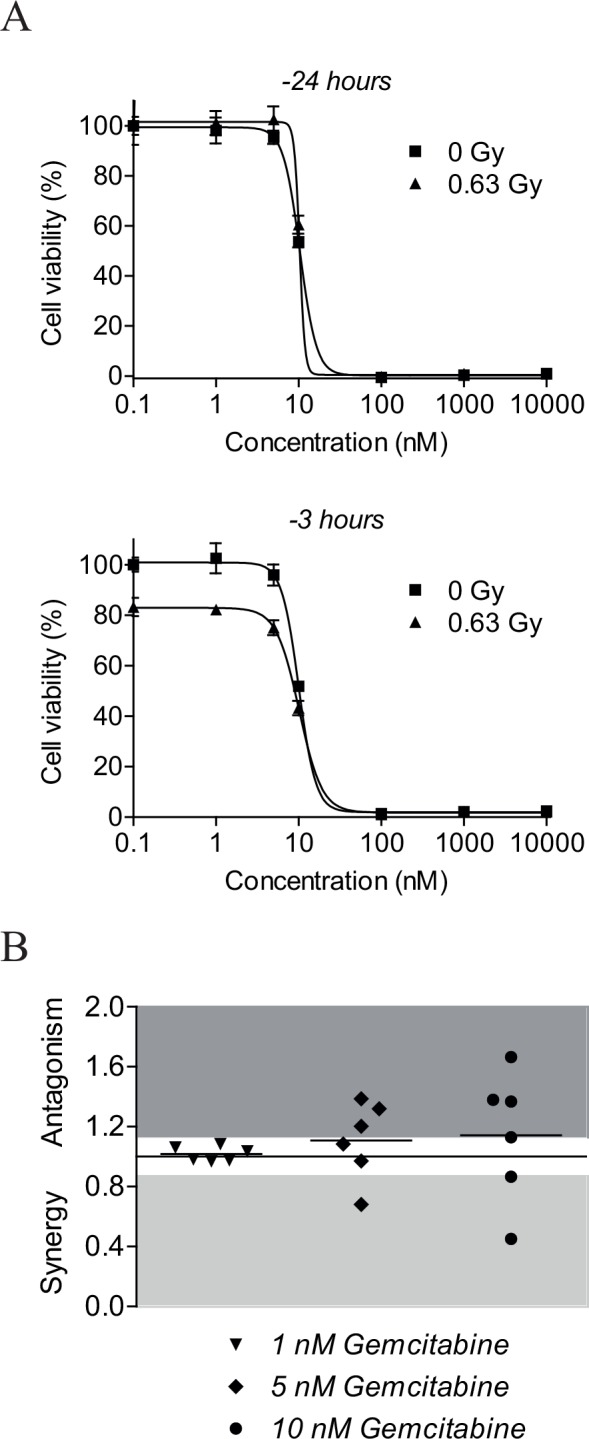
NU7026 more potently radiosensitizes neuroblastoma cells than gemcitabine. (A) Cell viability of NGP cells after co-treatment with gemcitabine and IR. Cells were pre-treated with 0, 1, 5, 10 or 100 nM or 1 or 10 μM gemcitabine for 3 or 24 h before exposure to 0.63 Gy IR. At 96 h after IR-exposure, MTT cell proliferation assays were performed to study effects on cell viability. Data represent the mean (n = 4) +/- SD. (B) Radiosensitizing effects of 1, 5 and 10 nM gemcitabine for neuroblastoma cell lines NGP, SHSY5Y, SHEP2, SJNB12, LAN5 and SKNBE(2). Cells were pre-treated with gemcitabine for 3 h before exposure to 0.63 Gy IR. At 96 h after IR-exposure, MTT cell proliferation assays were performed to assess cell viability (n = 4 for each cell line). Radiosensitizing effects were estimated by calculating combination indices (CI) according to Chou and Talalay [40], given on the Y-axis. CI > 1.1 is antagonistic (dark grey), 1.1 ≥ CI ≥ 0.9 is additive (white) and CI < 0.9 is synergistic (light grey). Horizontal lines between the symbols represent the mean combined effects.

Gemcitabine is a chemotherapeutic agent which is currently evaluated extensively in clinical trials for its additional radiosensitizing potential [41]. It is suggested that gemcitabine exerts its radiosensitizing effect via inhibition of ribonucleotide reductase by its active metabolite gemcitabine diphosphate, thereby reducing the synthesis of deoxynucleoside triphosphates [41]. Current study compared the radiosensitizing effects of NU7026 in neuroblastoma cell lines with the radiosensitizing effects of gemcitabine. As for NU7026, we first investigated the optimal gemcitabine dose for combination treatment with 0.63 Gy IR in NGP cells. Based on literature [41–43], NGP cells were pre-treated with 0, 1, 5, 10 or 100 nM or 1 or 10 μM gemcitabine for 3 or 24 h before irradiation. Effects on cell viability were studied at 96 h after IR-exposure of the cells. Twenty-four hours pre-treatment with gemcitabine did not improve the inhibitory effect of low-dose IR treatment on the cell viability of NGP cells, while 3 h pre-treatment did (Fig 5A). Monotherapy of NGP cells with ≥ 100 nM gemcitabine furthermore resulted into almost complete cell death (i.e. 97–99%). For the comparison with NU7026, gemcitabine was therefore administered to the cells in doses below 100 nM (i.e. 1, 5 and 10 nM) at 3 h before IR-exposure.

Radiosensitizing effects of gemcitabine were studied for the same neuroblastoma cell line panel as used for NU7026. While NU7026 synergistically radiosensitized all tested neuroblastoma cell lines to 0.63 Gy IR (i.e. mean CI = 0.60), only an additive effect on cell viability was observed for the combination between 1 nM gemcitabine and 0.63 Gy IR (i.e. 0.97 ≤ CI ≤ 1.08; mean CI = 1.02). Except for neuroblastoma cell line LAN5, higher doses of gemcitabine seemed to even result in less favorable effects (i.e. mean CI for 5 nM gemcitabine = 1.11; mean CI for 10 nM gemcitabine = 1.14) (Fig 5B). So in this experimental setup the DNA-PKcs inhibitor NU7026 exerted a more significant radiosensitizing effect in neuroblastoma cell lines than gemcitabine.

### Radiosensitization is DNA-PKcs dependent

**Fig 6:**
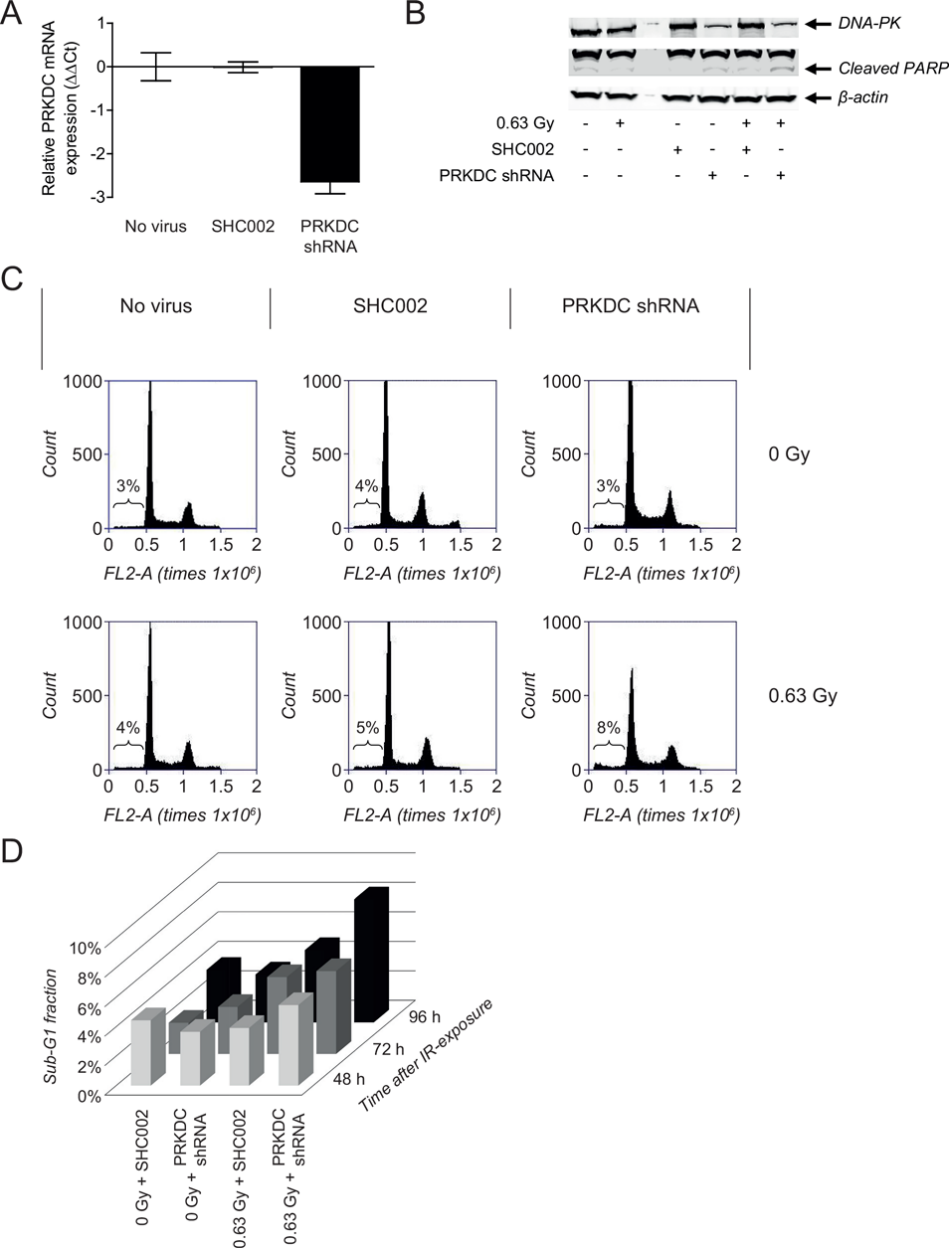
*PRKDC* knockdown in NGP cells confirms DNA-PKcs-dependent radiosensitizing activity of NU7026. (A) *PRKDC* mRNA expression in NGP cells at 144 h after transduction with nonspecific control shRNA (SHC002) or *PRKDC* shRNA. *PRKDC* mRNA levels were determined by qPCR and ΔCt values were normalized to the household gene β-actin. Normalized ΔCt values of untreated non-transduced NGP cells (no virus) were set at zero, giving ΔΔCt. Data represent the mean (n = 4) +/- SD. (B) FACS analysis of the effects of treatment with 0.63 Gy IR on the sub-G1 fraction of normal NGP cells and NGP cells transduced with SHC002 or *PRKDC* shRNA. Results are obtained at 96 h after IR-exposure of the cells. (C) Sub-G1 fraction of NGP cells transduced with SHC002 or *PRKDC* shRNA after non-treatment and after treatment with 0.63 Gy IR, at 48, 72 and 96 h after irradiation. (D) Western Blot analysis of protein levels of DNA-PKcs and cleaved PARP in non-irradiated and irradiated normal NGP cells and NGP cells transduced with SHC002 or *PRKDC* shRNA. Results were obtained at 120 h after irradiation, which was equal to 168 h after transduction. β-actin protein levels were used as loading control.

Small-molecule inhibitors, also when they are called specific, often possess off–target effects. To further investigate if the radiosensitizing effects observed for NU7026 are caused by DNA-PKcs inhibition, validation studies were performed using NGP cells transduced with a *PRKDC* shRNA or a nonspecific control shRNA (SHC002). *PRKDC* gene silencing was observed at 144 h after transduction of the NGP cells with *PRKDC* shRNA, while SHC002 did not affect the gene expression level of *PRKDC* (Fig 6A). Effects on gene level were confirmed on protein level by Western Blot analysis of DNA-PKcs (Fig 6B).

No apoptotic responses were observed after transduction of NGP cells with SHC002 or *PRKDC* shRNA, as shown by the lack of significant changes in sub-G1 fraction (Fig 6C). While only a 1% increase in sub-G1 fraction was observed after irradiation of SHC002-transduced NGP cells, exposure of *PRKDC* shRNA-transduced NGP cells to 0.63 Gy IR resulted in an increase in sub-G1 fraction of 4% at 96 h after irradiation (Fig 6C). As was seen for the combination of NU7026 and IR, the apoptotic response was more pronounced at 96 h than at 48 or 72 h after treatment (Fig 6D). Effects on sub-G1 fraction were confirmed by Western Blot analysis of PARP cleavage, with the highest cleaved PARP levels being observed after irradiation of NGP cells with *PRKDC* knockdown (Fig 6B).

Although the knockdown studies showed less potent effects, these results confirmed that the observed radiosensitizing effects of NU7026 are at least partially caused due to inhibition of its target DNA-PKcs.

## Discussion

This is the first manuscript reporting the *in vitro* radiosensitization of neuroblastoma cells by pre-treatment with a DNA-PKcs inhibitor. Radiosensitization of tumor cells by preventing NHEJ of DSBs has successfully been studied for different tumor types (e.g. breast cancer, ovarian cancer, colon cancer, cervical cancer and pancreatic cancer [29, 31, 44–46]), after it was shown that DSB repair by NHEJ is the predominant mechanism by which tumor cells might escape from radiation-induced cell death [8]. Currently, DNA-PKcs and DNA ligase IV are the only druggable targets in the NHEJ pathway [27]. As DNA-PKcs is one of the most upstream key players in NHEJ [27], it is not surprising that DNA-PKcs inhibitors are more frequently tested for their potency to radiosensitize tumor cells and overcome radioresistance than DNA ligase IV inhibitors. In the current manuscript, we studied the radiosensitizing potential of DNA-PKcs inhibitor NU7026 for neuroblastoma treatment.

Synergistic responses between IR and NU7026 were initially calculated based on cell viability assays which could consist of a growth inhibitory effect combined with a cell killing response. We showed that apoptosis-induced cell killing showed a much stronger synergistic response compared to the cell viability assays. This implies that neuroblastoma cells show a much stronger induction of cell death if radiation therapy is combined with a DNA-PKcs inhibitor and therefore a switch could be made from a cytostatic to a cytotoxic effect on neuroblastoma cells. Of note, irradiated cells might also be killed through mechanisms other than apoptosis, i.e. mitotic catastrophe, autophagy and necrosis [47]. These mechanisms of cell death have not been investigated in the current study.

Although promising, the increased risk of toxic effects on healthy tissues should be taken into account when adding a DSB repair inhibitor to radiation therapy. Strategies to restrict side effects on healthy tissues are limiting doses and/or using a radiosensitizer acting on a differentially expressed target in neuroblastoma tumors, resulting in a tumor-specific sensitizing effect. In determining the most optimal dose for combination treatment with NU7026 and IR, a balance was made between the effects obtained with NU7026 or IR alone or in combination. For example, 20 μM NU7026 more potently sensitized NGP cells to 0.63 Gy IR than 10 μM of the DNA-PKcs inhibitor, as shown by the CIs given in S1 Table. But dose escalation from 10 to 20 μM resulted into 19% more cytotoxicity of NU7026, while the gained combined anticancer activity was only 4% (i.e. 93 versus 89%). Similarly, equal combined effects could be obtained when combining lower NU7026 doses with higher IR doses, but in many of these cases weaker cytotoxic effects obtained with NU7026 were compensated by stronger cytotoxic effects of IR. Comparing our results with previously published results for other cancer types showed that NU7026 doses most potently sensitizing NGP cells to IR were comparable with NU7026 doses radiosensitizing ovarian, pancreatic and gastric tumor cells [29–31]. However, in the other studies similar NU7026 doses were combined with much higher IR doses (i.e. 2–5 Gy) [29–31]. We showed that exposure of NGP cells pre-treated with 10 μM NU7026 to 6.25 instead of 0.63 Gy IR only increased the combined inhibitory effect on cell viability from 89 to 95%, equal to the effect observed without NU7026 pre-treatment.

As NU7026 inhibits DNA-PKcs, elevated DNA-PKcs levels in cancer cells might contribute to a more tumor cell-specific radiosensitizing effect. DNA-PKcs is expressed in normal tissues such as colon, pancreas and kidneys [48]. However, various tumor types have been associated with elevated *PRKDC* mRNA- and/or DNA-PKcs levels [28, 49]. We demonstrated that also neuroblastoma express higher levels of *PRKDC* mRNA compared with normal tissues. Radiosensitizing effects of NU7026 were evaluated for neuroblastoma cell lines expressing different *PRKDC* mRNA levels, including cell lines with the lowest (i.e. SHSY5Y) and highest (i.e. NGP) *PRKDC* expression (S1 Fig). No direct correlation could be found between *PRKDC* mRNA levels and extent of radiosensitization. Synergistic radiosensitizing effects of NU7026 observed for all tested neuroblastoma cell lines indicate that even the lowest *PRKDC* mRNA level observed in neuroblastoma cell lines was high enough to induce radiosensitization. NU7026 did not radiosensitize low DNA-PKcs-expressing fast-proliferating fibroblasts that share some of their characteristics with cancer cells. Future studies need to focus on the *PRKDC* mRNA cutoff values above which NU7026 radiosensitizes neuroblastoma cells and on the correlation between *PRKDC* mRNA levels and DNA-PKcs protein levels in neuroblastoma.

NU7026 is one of the first DNA-PKcs inhibitors derived from the nonspecific DNA-PKcs inhibitor LY294002 to obtain a more potent inhibitor with improved specificity towards DNA-PKcs [8, 26]. Due to similarities between the kinase domains of DNA-PKcs and PI3K, LY294002 potently inhibits both kinases [8]. NU7026 inhibits DNA-PKcs much more potently than PI3K with IC_50_ values of 0.23 μM and 13 μM, respectively [26, 27, 31]. Currently, LY294002 has been used as a backbone for the development of several other DNA-PKcs inhibitors including NU7441 [8, 26]. NU7441 is more potent and specific than NU7026 with IC_50_ values against DNA-PKcs and PI3K of 14 nM and 5 μM, respectively, [49, 50] and possesses more favorable pharmacokinetic characteristics leading to an improved residence time in the tumor [26]. Together with its radiosensitizing effects observed in pre-clinical studies for other cancer types [45, 46, 51], these properties make NU7441 a valuable compound for adjuvant testing in neuroblastoma cells. At this moment, NU7026 and NU7441 not yet reached clinical phase.

Results obtained with the small-molecule inhibitor NU7026 were confirmed by *PRKDC* knockdown, proving that NU7026 radiosensitizes neuroblastoma cells due to DNA-PKcs inhibition. But the radiosensitizing effect of *PRKDC* knockdown was less potent than the radiosensitizing effects of NU7026. This might relate to incomplete DNA-PKcs silencing or the fact that NU7026 inhibits additional targets [31, 52, 53].

Taken together, this paper demonstrates that DNA-PKcs inhibition synergistically sensitized neuroblastoma cells to low radiation doses. Combination therapy with 10 μM NU7026 and 0.63 Gy IR resulted in one of the most favorable effects, leading to the effective killing of neuroblastoma cells. In contrast, NU7026 did not radiosensitize low DNA-PKcs expressing non-cancerous fibroblasts. Together with the observation that neuroblastoma patients have increased levels of the gene encoding for DNA-PKcs (i.e. *PRKDC*), these results make DNA-PKcs a promising target for neuroblastoma radiosensitization.

## Supporting Information

S1 Fig *PRKDC* mRNA expression levels in neuroblastoma cell lines. *PRKDC* mRNA expression levels were analyzed by Affymetrix microarrays. Black symbols represent the expression levels in the neuroblastoma cell lines included in the current study.
(TIF)

S2 Fig NU7026 radiosensitizes high DNA-PKcs-expressing NGP cells. (A) Effects of NU7026 plus IR combination therapy versus monotherapy on the colony forming capacity of NGP cells. Cells in 0.4% agar in culture medium were seeded on top of a hardened 0.5% agar base layer. After overnight incubation at normal culture conditions, cells were 1 h pre-incubated with 0, 2, 10 or 20 μM NU7026 in 0.4% agar in culture medium before exposure to 0 or 0.63 Gy IR (n = 3 per condition). The following 3 weeks, fresh DMSO or NU7026 in 0.4% agar in culture medium was added to the cells once a week. Colonies were subsequently visualized by 4 h incubation with MTT. (B) Western Blot analysis of DNA-PKcs protein levels in neuroblastoma cell lines NGP and SKNBE(2) and fibroblast cell lines F2112 and F1366. α-Tubulin protein levels were used as loading control. Separate analysis of the fibroblast cell lines showed that the non-cancerous fast-proliferating fibroblast cell lines F2112 and F1366 express low levels of DNA-PKcs (right pictures).
(TIF)

S1 Table Sensitivity of NGP cells to NU7026 plus IR combination therapy versus monotherapy. Percentage inhibition of the cell viability after monotherapy or combination therapy of NGP cells with indicated doses of NU7026 and/or IR. Combination indices (CIs) are given between brackets and calculated according to Chou and Talalay [40]. CI > 1.1 is antagonistic, 1.1 ≥ CI ≥ 0.9 is additive and CI < 0.9 is synergistic.
(DOCX)
