# Supplementary figures and images for: DNA-Dependent Protein Kinase As Molecular Target for Radiosensitization of Neuroblastoma Cells
Source: PLoS One. 2015 Dec 30;10(12):e0145744. doi: 10.1371/journal.pone.0145744 (PMC4696738; doi:10.1371/journal.pone.0145744)

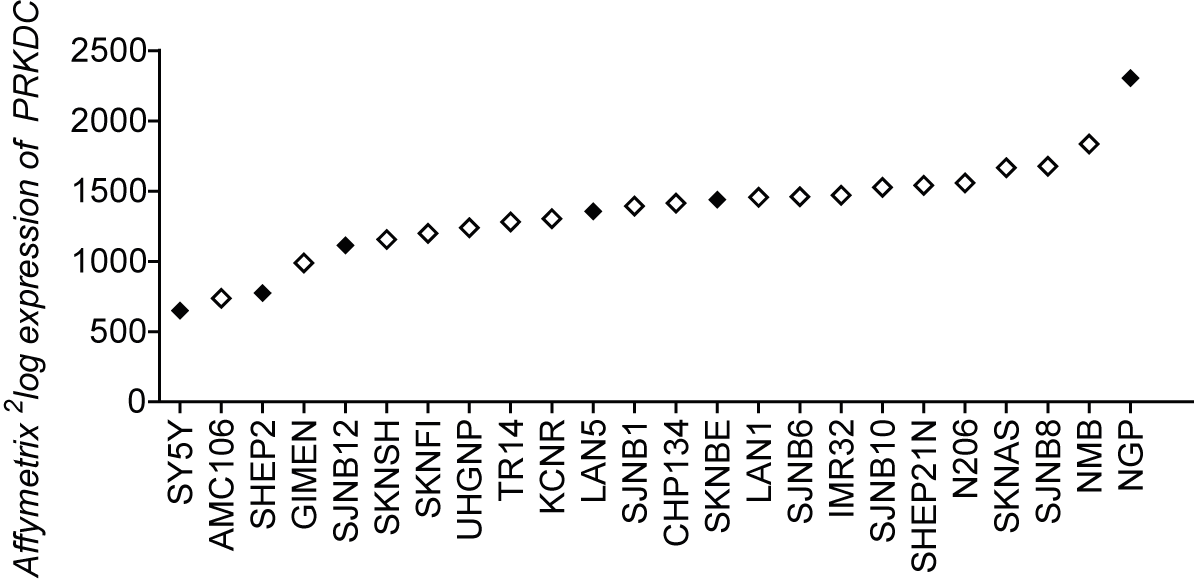

Supplement: S1 Fig — PRKDC mRNA expression levels were analyzed by Affymetrix microarrays. Black symbols represent the expression levels in the neuroblastoma cell lines included in the current study. (TIF) [file pone.0145744.s001.tif]

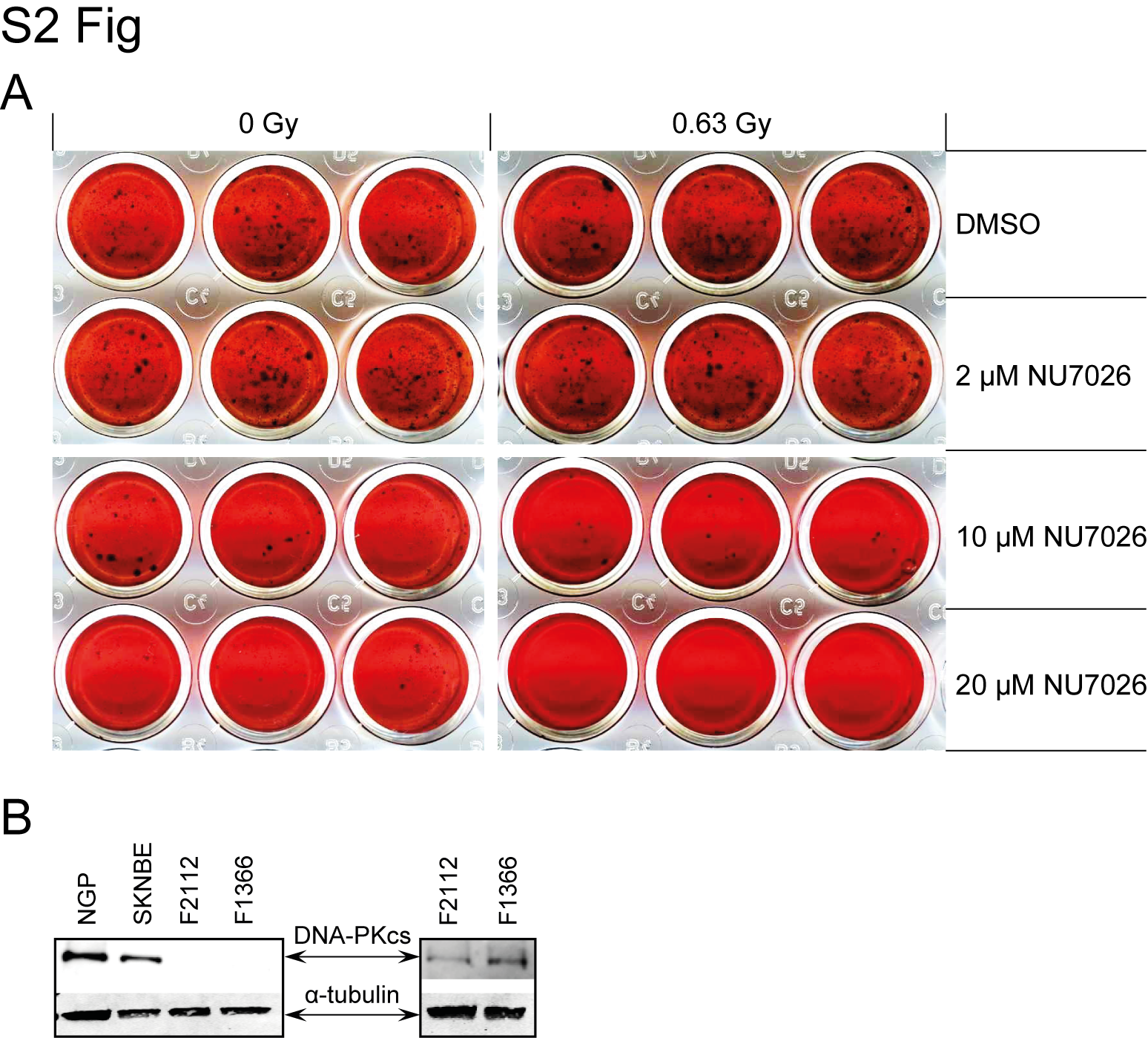

Supplement: S2 Fig — (A) Effects of NU7026 plus IR combination therapy versus monotherapy on the colony forming capacity of NGP cells. Cells in 0.4% agar in culture medium were seeded on top of a hardened 0.5% agar base layer. After overnight incubation at normal culture conditions, cells were 1 h pre-incubated with 0, 2, 10 or 20 μM NU7026 in 0.4% agar in culture medium before exposure to 0 or 0.63 Gy IR (n = 3 per condition). The following 3 weeks, fresh DMSO or NU7026 in 0.4% agar in culture medium was added to the cells once a week. Colonies were subsequently visualized by 4 h incubation with MTT. (B) Western Blot analysis of DNA-PKcs protein levels in neuroblastoma cell lines NGP and SKNBE(2) and fibroblast cell lines F2112 and F1366. α-Tubulin protein levels were used as loading control. Separate analysis of the fibroblast cell lines showed that the non-cancerous fast-proliferating fibroblast cell lines F2112 and F1366 express low levels of DNA-PKcs (right pictures). (TIF) [file pone.0145744.s002.tif]
