# Supplementary material for: DNA-Dependent Protein Kinase As Molecular Target for Radiosensitization of Neuroblastoma Cells
Source: PLoS One. 2015 Dec 30;10(12):e0145744. doi: 10.1371/journal.pone.0145744 (PMC4696738; doi:10.1371/journal.pone.0145744)
Supplement: S1 Table — Percentage inhibition of the cell viability after monotherapy or combination therapy of NGP cells with indicated doses of NU7026 and/or IR. Combination indices (CIs) are given between brackets and calculated according to Chou and Talalay [40]. CI > 1.1 is antagonistic, 1.1 ≥ CI ≥ 0.9 is additive and CI < 0.9 is synergistic. (DOCX) [file pone.0145744.s003.docx]

**S1 Table. Sensitivity of NGP cells to NU7026 plus IR combination therapy versus monotherapy.**

| **NU7026 (μM)**  **IR**  **(Gy)** | **0** | **2** | **5** | **10** | **20** |
| --- | --- | --- | --- | --- | --- |
| **0** | 0 | 4 | 20 | 31 | 50 |
| **0.12** | 19 | 29  (0.91) | 40  (0.89) | 55  (0.76) | 72  (0.63) |
| **0.25** | 36 | 44  (0.90) | 56  (0.80) | 70  (0.60) | 85  (0.38) |
| **0.37** | 38 | 53  (0.78) | 65  (0.65) | 80  (0.41) | 89  (0.29) |
| **0.50** | 51 | 63  (0.77) | 75  (0.57) | 87  (0.32) | 91  (0.27) |
| **0.63** | 46 | 58  (0.80) | 75  (0.53) | 89  (0.25) | 93  (0.20) |
| **1.25** | 52 | 70  (0.64) | 90  (0.23) | 94  (0.15) | 94  (0.19) |
| **1.88** | 66 | 82  (0.54) | 92  (0.26) | 94  (0.20) | 94  (0.24) |
| **2.50** | 79 | 89  (0.53) | 96  (0.20) | 95  (0.26) | 95  (0.29) |
| **6.25** | 95 | 97  (0.60) | 96  (0.81) | 95  (1.02) | 98  (0.42) |

Percentage inhibition of the cell viability after monotherapy or combination therapy of NGP cells with indicated doses of NU7026 and/or IR. Combination indices (CIs) are given between brackets and calculated according to Chou and Talalay [40]. CI > 1.1 is antagonistic, 1.1 ≥ CI ≥ 0.9 is additive and CI < 0.9 is synergistic.
